# Social Appearance Anxiety Scale: a psychometric investigation and evaluation of the influence of individual characteristics on social appearance anxiety in Brazilian adults who practice physical exercise

**DOI:** 10.3389/fpsyg.2023.1261605

**Published:** 2023-11-30

**Authors:** Giovanna Soler Donofre, Juliana Alvares Duarte Bonini Campos, Priscila Carvalho dos Santos, João Marôco, Lucas Arrais Campos, Wanderson Roberto da Silva

**Affiliations:** ^1^Department of Biological Sciences, School of Pharmaceutical Sciences, São Paulo State University (UNESP), São Paulo, Brazil; ^2^William James Center for Research (WJCR), University Institute of Psychological, Social, and Life Sciences (ISPA), Lisbon, Portugal; ^3^Flu Pedagogy, Nord University, Bodø, Norway; ^4^Faculty of Medicine and Health Technology, Tampere University, Tampere, Finland; ^5^Department of Ear and Oral Diseases, Tampere University Hospital, Tampere, Finland; ^6^School of Dentistry, São Paulo State University (UNESP), São Paulo, Brazil; ^7^Postgraduate Program in Food, Nutrition, and Food Engineering, São Paulo State University (UNESP), Araraquara, São Paulo, Brazil

**Keywords:** anxiety, physical appearance, validation study, population characteristics, structural equation modeling

## Abstract

**Introduction:**

Evaluating signs of anxiety related to body appearance is becoming increasingly important in contemporary society and, in this sense, the Social Appearance Anxiety Scale (SAAS) seems an interesting alternative of measurement.

**Objectives:**

To evaluate the psychometric properties of the Portuguese version of the SAAS when applied to Brazilian adults who practice physical exercise and verify the influence of individual characteristics on participants’ social appearance anxiety.

**Methods:**

This was a cross-sectional study conducted online. The participants completed the SAAS and a demographic questionnaire. The psychometric properties of the SAAS one-factor model were evaluated using confirmatory factor analysis. A structural model was built for men and women to verify the influence of individual characteristics of the participants on social appearance anxiety.

**Results:**

1,495 individuals participated in the study (70.8% women; mean age = 29.5, SD = 8.9 years). The data obtained with the SAAS presented good indicators of validity and reliability for both genders (CFI > 0.97, TLI > 0.97, SRMR = 0.04, α > 0.97, ω > 0.85). For both men and women, greater levels of social appearance anxiety were observed among younger participants, who had a higher body mass index, self-reported an eating disorder, and perceived a change in their body after the onset of the COVID-19 pandemic. For women specifically, higher income and having started physical exercise more recently were associated with greater levels of social appearance anxiety.

**Conclusion:**

The findings supported the validity and reliability of the data obtained with the SAAS and revealed that when investigating social appearance anxiety in future research and clinical protocols, specific individual characteristics should be considered.

## 1 Introduction

Anxiety can be characterized by apprehension and somatic symptoms of tension, in which an individual anticipates imminent danger or a catastrophe, causing a series of bodily adaptations to occur to mobilize the subject in the face of this possible situation. This emotion is understood as a future-oriented, long-acting, and broadly focused response to a threat [American Psychiatric Association (APA), 2013]. When anxiety exists excessively or persists beyond appropriate periods for the individual’s developmental level, an anxiety disorder can occur, with behaviors that can harm the individual’s life, such as social avoidance [American Psychiatric Association (APA), 2013]. When anxiety is related to the negative judgment of others about the appearance of one’s own body, a state of social physical anxiety is experienced (Hart et al., 1989).

The concept of social physique anxiety emerged in the 1980s when Hart et al. (1989) investigated the degree of anxiety that people have during physical assessment in gyms in a North American context. These authors described this concept as a state of body-related anxiety in response to potentially real and imaginary judgments from other individuals. To analyze signs and symptoms suggestive of social physique anxiety, Hart et al. (1989) developed the Social Physique Anxiety Scale (SPAS). The SPAS is a psychometric scale composed of 12 items and has been used in different contexts (Hagger et al., 2007; Neves et al., 2018). However, psychometric evidence indicates the fragility of the factorial model of SPAS (da Silva et al., 2022). Therefore, the use of other scales can be interesting to obtain valid and reliable data on social anxiety and physical appearance. Based on that, Hart et al. (2008) proposed the Social Appearance Anxiety Scale (SAAS). This scale assesses social appearance anxiety, which is a broader concept than that assessed by the SPAS. The SAAS items encompass content related to the fear that an individual has that their appearance will be evaluated by other people and that this will harm their life in different contexts (e.g., job opportunities and peer relationships). Therefore, the SAAS includes content that goes beyond a body concern (Hart et al., 2008).

The SAAS has 16 items, written as statements, with a 5-point Likert-type response scale (Hart et al., 2008). The scale has already been translated and cross-culturally adapted for different countries (Hart et al., 2008; Claes et al., 2012; Dakanalis et al., 2016; Radix et al., 2018). In addition, studies have shown adequate psychometric properties of this scale, suggesting its good ability to screen signs and symptoms of social appearance anxiety (Claes et al., 2012; Papapanou et al., 2023). Donofre et al. (2021) developed a Portuguese version of the SAAS for the Brazilian context, but its psychometric properties have not yet been evaluated. It would be important to attest the validity and reliability of the data obtained with this version, considering that the Brazilian population is largely concerned with body appearance (Santos Silva et al., 2011). This is probably related to the pursuit of a socially accepted body standard, which has gained emphasis since the beginning of the 21st century (Witt and Schneider, 2011). Thus, using the SAAS to screen for social appearance anxiety becomes important. However, conducting psychometric evaluation is crucial to ensure the validity and reliability of the data obtained by the scale, serving as a necessary methodological step before intervention studies (Marôco, 2021).

Understanding the relationship between social appearance anxiety and individual characteristics is also relevant to develop appropriate scientific and clinical protocols. Relationships have been found between higher scores of social appearance anxiety and younger age, as well as unemployment (Papapanou et al., 2023). Characteristics such as body mass index and body fat and their relationships with social appearance anxiety have also been studied (Levinson and Rodebaugh, 2011). Therefore, it seems interesting to verify how similar characteristics relate to appearance anxiety in other contexts. The experience of a current or previous eating disorder may also be related to social appearance anxiety, as people with this condition suffer constant disturbances in body image and have difficulty engaging in social relationships [American Psychiatric Association (APA), 2013]. During the COVID-19 pandemic, people’s routine changed significantly and this may have influenced the perception and possibly dissatisfaction of their body appearance (Castro et al., 2021), possibly contributing to greater social anxiety. Furthermore, some studies (Brunet and Sabiston, 2009; Baillot et al., 2020; Herring et al., 2021) have explored the relationships between characteristics of physical exercise (e.g., intensity and frequency) and social physique anxiety. A recent study conducted with the Italian population aimed to investigate the relationship between autonomous motivation and physical activity (Galli et al., 2022). The study examined the role of behavioral intention and anxiety in this context. The findings revealed that anxiety, depending on its levels, might increase the influence of the intention to engage in physical exercise, particularly within the context of COVID-19. These studies demonstrate the importance of better understanding these issues, particularly because people who engage in physical exercise may be a population with more concerns related to appearance and greater anxiety in general.

Given the above, this study was conducted with two aims. The first aim was to evaluate the psychometric properties of the Portuguese version of the SAAS when applied to Brazilian adults who practice physical exercise. Given the evidence that the SAAS one-factor model (original) presents adequate parameters of validity and reliability in different contexts, the hypothesis of the present study was that this model would also present good psychometric indicators in the Brazilian context. The second aim was to verify the influence of participants’ characteristics on social appearance anxiety. For that, a structural model was elaborated, which included the characteristics mentioned in the literature as relevant to social appearance anxiety. This model was tested for men and women separately, as these groups have different experiences in relation to body image, which is already well explained in the literature (Cash et al., 2004; Griffiths et al., 2016). For the second aim, the hypothesis was that younger participants, having a higher socioeconomic level, possessing a higher BMI, practicing physical exercises for longer, having already had an eating disorder (self-report), and reporting having noticed a change in the body image after the onset of the COVID-19 pandemic would present greater social appearance anxiety.

## 2 Methods

### 2.1 Study design and sampling method

This was a cross-sectional study with non-probabilistic sampling. The minimum sample size was estimated using the recommendation of 10 participants for each parameter estimated in the factorial model of the SAAS and in the final structural model (Hair et al., 2019). A total of 38 parameters was estimated, and a loss rate of 10% also was added; therefore, the minimum sample size was 423 individuals. As the analyses were performed separately for men and women, this minimum sample size was designed for each gender.

### 2.2 Participants and procedures

Brazilian adults who self-reported practicing physical exercise – including those performed at home, in a public environment such as parks or squares, or at gyms – participated in the study. Inclusion criteria were age between 18 and 59 years old and exercising for at least 3 months. Exclusion criteria were pregnant or lactating women. To ensure the eligibility of participants, a screening questionnaire was used before the definitive data collection.

Data collection was conducted from May to August of 2021 through a non-probabilistic online survey using Google Forms® (Alphabet Inc., CA, USA). When accessing the survey, participants had to be logged in with an e-mail to limit one response per person. Participants were invited to voluntarily complete the survey through various channels, aiming to obtain a broad and diverse sample. Initially, the invitations were sent from the researchers’ email addresses to the directors and coordinators of several undergraduate and graduate courses at state, federal, and private universities in Brazil. This strategy especially reached the academic community (i.e., students and professors). Additionally, the research was also promoted through WhatsApp groups and Instagram profiles, where the research aims, and participation process were shared. When individuals agreed to participate, they were asked to promote the study to other people through their social networks and individual groups, adopting a snowball sampling strategy.

All individuals who agreed to participate first voluntarily accessed the link to the survey and signed the informed consent form (first page). Afterward, the participants completed the items for sample characterization (demographic questionnaire), followed by the SAAS and the SPAS. This study followed the guidelines of the Declaration of Helsinki and was approved by the Research Ethics Committee of the Faculty of Pharmaceutical Sciences of Araraquara (UNESP) (CAAE: 22051619.8.0000.5426).

### 2.3 Measures

#### 2.3.1 Sample characterization

A demographic questionnaire was developed by the authors to include data on age, gender (“How do you identify?”: male; female; transgender; non-binary; others; and prefer not to answer), region of Brazil residing (North; Northeast; Central-west; South; and Southeast), marital status (single; married; separate; and divorced), race (white; black; biracial or multiracial; Asian; indigenous; and prefer not to inform), level of education (complete primary education; complete high school; complete higher education; and complete postgraduate), work activity (no; yes), monthly family income (< R$ 1.254,00; R$ 1.255,00 to R$ 2.004,00; R$ 2.005,00 to R$ 8.640,00; R$ 8.641,00 to R$11.261,00; > R$ 11.262,00 – the exchange rate in October 2022 was 1 U$ dollar to 5.27 reais[R$]), weight and height, how long have been exercising (less than 6 months; from 6 months to 1 year; from 1 year to 1 year and a half; from 1 year and a half to 2 years; more than 2 years) and weekly frequency (1 to 7 or more), and if they were a professional athlete (no; yes). Having already received a medical diagnosis of an eating disorder (no; yes) and self-perception of changes in body appearance and exercise practice after the onset of the COVID-19 pandemic (not even a little; lightly; a little bit; much; extremely) were also included.

#### 2.3.2 Social Appearance Anxiety Scale (SAAS)

This scale was originally proposed in English by Hart et al. (2008) to assess social appearance anxiety as a one-factor model. SAAS has 16 items (e.g., “I feel nervous when having my picture taken”). The first (“I feel comfortable with the way I appear to others”) is inverted in relation to the others. Response scale ranges from not at all (1) to extremely (5), with higher scores indicating greater social appearance anxiety. Participants completed the Portuguese version of the SAAS (Donofre et al., 2021).

#### 2.3.3 Social Appearance Anxiety Scale (SAAS)

This scale was originally proposed in English by Hart et al. (1989) to assess social physique anxiety as a one-factor model. SPAS had 12 items, seven of which are negative statements and five are positive ones. The response scale is presented in a 5-point Likert-type scale ranging from not at all (1) to extremely characteristic (5). After reversing the responses to regular items (for a review, see da Silva et al., 2022) a higher score indicated greater social physical anxiety. Participants completed a Portuguese version of the SPAS, in which all items are written in the same direction (i.e., negative statements) (da Silva et al., 2022).

#### 2.3.4 Godin-shephard leisure-time physical activity questionnaire (GSLTPAQ)

The questionnaire was proposed by Godin and Shephard (1985) to differentiate between sedentary and active individuals. GSLTPAQ has two questions. The first question has three sub-items where individuals indicate the number of times they practice physical activities for at least 15 min at (i) vigorous, (ii) moderate, and (iii) light intensity, considering 7 days per week. The second question measures the frequency of leisure-time activities that cause sweating. To calculate the final score, the frequency indicated by the participant in the first question is multiplied by an effort coefficient ([9 × vigorous] + [5 × moderate] + [3 × light]). Higher scores indicate a higher level of physical activity. The participants completed the Portuguese version of the GSLTPAQ (Sao-Joao et al., 2013).

### 2.4 Data analysis

Analyzes were performed separately for men and women. First, mean, median, mode, standard deviation, skewness, and kurtosis were calculated for all SAAS items to verify the distribution of participants’ responses and identify the psychometric sensitivity of the data. Absolute values of skewness and kurtosis lower than 3 and 7, respectively, were indicative of a non-severe violation of the normal distribution of the data (Marôco, 2021).

The factorial validity of the SAAS one-factor model was estimated using Confirmatory Factor Analysis (CFA). The Weighted Least Squares Mean and Variance Adjusted (WLSMV) estimation method was used. The goodness-of-fit indices used in CFA were the Comparative Fit Index (CFI), Tucker-Lewis Index (TLI), and Standardized Root Mean Square Residual (SRMR) (Fornell and Larcker, 1981; Byrne, 2010). Values of CFI and TLI values ≥0.95 and SRMR <0.07 were indicative of adequate factorial validity of the model (Hu and Bentler, 1999; Hair et al., 2019). The factor loading (*λ*) of each SAAS item was calculated and those with values greater than 0.50 were kept in the model. To assess convergent validity, that is, the adequacy of the items for the composition of the factor, the average variance extracted (AVE) was estimated from the values of the factor loadings (Fornell and Larcker, 1981). Values of AVE ≥ 0.50 indicated adequate convergent validity (Fornell and Larcker, 1981). To assess concurrent validity, which is the relationship between latent concepts, a correlational analysis was conducted between the one-factor model of the SAAS (Hart et al., 2008) and the one-factor model of the SPAS (da Silva et al., 2022). In this analysis, we hypothesized that both scales, which assess related concepts, would present a strong and significant correlation (*p* < 0.05).

The reliability was assessed using the ordinal alpha (*α*) and omega (*ω*) coefficients and values ≥0.70 indicated adequacy (Zumbo et al., 2007; Gadermann et al., 2012). To assess the equivalence of the one-factor model of the SAAS across independent subsamples, measurement invariance tests were performed using multigroup analysis (Chen, 2007). For that, the sample of each gender (men: *n* = 426, women: *n* = 1,058) was randomly split into two parts and the analysis was performed for men (subsample 1: *n* = 213 vs. subsample 2: *n* = 213) and women (subsample 1: *n* = 529 vs. subsample 2: *n* = 529). Initially, the configural invariance (baseline model) was guaranteed. Subsequently, the equivalence of factor loadings (metric model), thresholds (scalar model), and residuals (residual model) was evaluated, which were compared two by two through the difference (Δ) in the CFI values. When a reduction in CFI values < −0.01 was observed, the one-factor SAAS model showed strong invariance (Chen, 2007).

The last step consisted of verifying the influence of individual characteristics on participants’ social appearance anxiety. For that, a structural model was built for each gender using the structural equation modeling. In both models, the independent variables were age (continuous), monthly income (1: <R$1,254; 2: R$1,255–2,004; 3: R$2,005–8,640; 4: R$8,641–11,261; 5: >R$11,262.00), BMI (continuous), length of exercising (1: <6 months, 2: 6 months to 1 year, 3: 1 to 1.5 year, 4: 1.5 to 2 years, 5: >2 years), self-reported diagnosis of an eating disorder (0: no, 1: yes), and self-report of change in body appearance during the COVID-19 pandemic (1: not at all, 2: slightly, 3: a little, 4: a lot, 5: extremely). The pathways (*β*) were analyzed and compared to the critical ratios of z (Marôco, 2021). A significance level of 5% was used. The fit of the models was assessed using CFI, TLI, and SRMR indices considering the previously mentioned adequacy values (Hu and Bentler, 1999). The analyses were performed using the R software (v. 4.1.3), with RStudio (v. 2022.02.0 + 443), using the foreign, lavaan, SemTools, and psych packages.

## 3 Results

A total of 1,524 individuals completed the survey; however, 29 did not complete all the SAAS items and were excluded from the analyses. Thus, the final sample was 1,495 individuals, of which 70.8% identified themselves as woman, 28.5% as men, 0.3% as non-binary, 0.3% chose the category others, and 0.1% chose not to respond. Considering that most identified themselves as man or woman, the analyses were separated for these groups and the others were excluded from the psychometric analyses, as the sample size was too small to be used. These individuals were included in the descriptive analyses referring to the total sample.

The mean age for the total sample was 29.5 (SD = 8.9) years (men: *M* = 30.0, SD = 9.4 years; women: *M* = 29.4, SD = 8.7 years). The mean BMI of the total sample was 24.7 (SD = 4.7) kg/m^2^ (men: *M* = 25.7, SD = 4.0 kg/m^2^; women: *M* = 24.4, SD = 4.9 kg/m^2^). GSLTPAQ data showed that 87.8% of the participants were physically active (92.7% men, 85.7% women), 8.8% were moderately active (6.1% men, 9.9% women) and 3.4 insufficiently active (1.2% men, 4.3% women). No participant was sedentary, as this was an exclusion criterion.

Table 1 presents the characterization of the sample. Most participants indicated that they live in the Southeastern region of Brazil, are white, have a high level of education, have been exercising for over 2 years, and reported not having received a medical diagnosis of an eating disorder. According to BMI, most participants were eutrophic. Most reported that they noticed a change in their own body appearance after the onset of the COVID-19 pandemic and that change was a little worse and promoted dissatisfaction. For physical exercise, most participants reported that the practice was very modified after the onset of the COVID-19 pandemic and that this change was a little worse and promoted dissatisfaction.

**Table 1:** Sample characterization.

	Sample *n* (%)		
Characteristic	Total (*n* = 1495)	Men (*n* = 426)	Women (*n* = 1058)
**Region of Brazil where reside**			
North	39 (2.6)	10 (2.3)	29 (2.7)
Northeast	133 (8.9)	42 (9.9)	89 (8.4)
Central-west	83 (5.6)	19 (4.5)	64 (6.0)
South	158 (10.6)	34 (8.0)	124 (11.7)
Southeast	1082 (72.4)	321 (75.4)	752 (71.1)
**Marital status**			
Single	997 (66.9)	287 (67.7)	701 (66.4)
Married	451 (30.3)	130 (30.7)	320 (30.3)
Separate	41 (2.8)	7 (1.7)	34 (3.2)
Divorced	1 (0.1)	–	1 (0.2)
**Race**			
White	1146 (76.6)	291 (68.3)	848 (80.2)
Black	70 (4.7)	26 (6.1)	43 (4.1)
Mixed	220 (14.7)	90 (21.1)	128 (12.1)
Asian	33 (2.2)	7 (1.6)	26 (2.5)
Indigenous (native)	3 (0.2)	2 (0.5)	1 (0.1)
Prefer not to inform	23 (1.5)	10 (2.3)	12 (1.1)
**Level of education**			
Complete primary education	4 (0.3)	–	3 (0.3)
Complete high school	475 (31.8)	148 (34.8)	322 (30.4)
Complete higher education	437 (29.3)	130 (30.6)	303 (28.6)
Complete postgraduate	577 (38.6)	147 (34.6)	430 (40.6)
**Work activity**			
No	454 (30.4)	120 (28.2)	327 (30.9)
Yes	1040 (69.6)	305 (71.8)	731 (69.1)
Monthly family income*			
< R$ 1.254,00	52 (3.5)	18 (4.2)	32 (3.0)
R$ 1.255,00 to R$ 2.004,00	127 (8.5)	32 (7.5)	94 (8.9)
R$ 2.005,00 to R$ 8.640,00	728 (48.9)	217 (51.1)	506 (48.0)
R$ 8.641,00 to R$11.261,00	237 (15.9)	67 (15.8)	168 (15.9)
> R$ 11.262,00	345 (23.2)	91 (21.4)	254 (24.1)
**Have you ever received a medical diagnosis of an eating disorder?**			
No	1369 (91.9)	416 (98.3)	945 (89.5)
Yes	121 (8.1)	7 (1.7)	111 (10.5)
**Do you believe that the appearance of your body has changed since the beginning of the pandemic (COVID-19)?**			
Not even a little	145 (9.7)	65 (15.3)	79 (7.5)
Lightly	293 (19.6)	95 (22.3)	195 (18.4)
A little bit	617 (41.3)	163 (38.3)	451 (42.6)
Much	314 (21.0)	70 (16.4)	241 (22.8)
Extremely	126 (8.4)	33 (7.7)	92 (8.7)
**If there was a change, this was for:**			
Much worse	122 (8.8)	25 (6.6)	95 (9.5)
A little worse	532 (38.2)	125 (32.9)	406 (40.5)
Almost the same	268 (19.3)	89 (23.4)	176 (17.6)
A little better	349 (25.1)	102 (26.8)	244 (24.4)
Much better	121 (8.7)	39 (10.3)	81 (8.1)
**Your satisfaction with this change was:**			
Very dissatisfied	153 (10.9)	29 (7.6)	121 (12.0)
Dissatisfied	487 (34.8)	111 (29.1)	375 (37.2)
Neutral	355 (25.3)	118 (30.9)	233 (23.1)
Satisfied	335 (23.9)	101 (26.4)	231 (22.9)
Very satisfied	71 (5.1)	23 (6.0)	48 (4.8)
**How long have you been exercising?**			
Less than 6 months	255 (17.1)	60 (14.2)	194 (18.4)
From 6 months to 1 year	177 (11.9)	39 (9.2)	135 (12.8)
From 1 year to 1 year and a half	109 (7.3)	22 (5.2)	84 (7.9)
From 1 year and a half to 2 years	77 (5.2)	24 (5.7)	53 (5.0)
More than 2 years	874 (58.6)	279 (65.8)	591 (55.9)
**Frequency of physical exercise (times/week)**			
1	33 (2.2)	13 (3.1)	20 (1.9)
2	209 (14.0)	39 (9.2)	168 (15.9)
3	421 (28.2)	117 (27.5)	301 (28.4)
4	287 (19.2)	70 (16.4)	216 (20.4)
5	334 (22.4)	111 (26.1)	220 (20.8)
6	160 (10.7)	56 (13.1)	103 (9.7)
7 or more	50 (3.3)	20 (4.7)	30 (2.8)
**Are you a professional athlete?**			
No	1469 (98.3)	417 (97.9)	1041 (98.4)
Yes	26 (1.7)	9 (2.1)	17 (1.6)
**Do you believe that your practice of physical exercise has changed due to the pandemic (COVID-19)?**			
Not even a little	80 (5.4)	29 (6.8)	50 (4.7)
Lightly	172 (11.5)	56 (13.1)	113 (10.7)
A little bit	437 (29.2)	123 (28.9)	313 (29.6)
Much	511 (34.2)	136 (31.9)	373 (35.3)
Extremely	295 (19.7)	82 (19.2)	209 (19.8)
**If there was a change, this was for:**			
Much worse	293 (20.4)	85 (20.9)	207 (20.3)
A little worse	506 (35.2)	160 (39.3)	340 (33.4)
Almost the same	193 (13.4)	66 (16.2)	124 (12.2)
A little better	231 (16.1)	46 (11.3)	185 (18.2)
Much better	213 (14.8)	50 (12.3)	162 (15.9)
**Your satisfaction with this change was:**			
Very dissatisfied	201 (13.9)	51 (12.4)	121 (12.0)
Dissatisfied	524 (36.3)	152 (36.9)	375 (37.2)
Neutral	291 (20.1)	115 (27.9)	233 (23.1)
Satisfied	285 (19.7)	69 (16.7)	231 (22.9)
Very satisfied	144 (10.0)	25 (6.1)	48 (4.8)
**Anthropometric nutritional status**			
Underweight	43 (2.9)	6 (1.4)	36 (3.4)
Eutrophic	877 (58.7)	205 (48.1)	664 (62.8)
Overweight	401 (26.8)	169 (39.7)	231 (21.9)
Obesity	173 (11.6)	46 (10.8)	126 (11.9)

*Monthly family income is presented in Brazilian reals (R$) and 1 US dollar is equivalent to 5.26 Brazilian reais according to the conversion rate in October 2022.

Table 2 presents the descriptive analysis of the responses given to the SAAS items. No item showed extreme values for the skewness and/or kurtosis in any of the samples, assuming there was psychometric sensitivity of the data.

**Table 2:** Descriptive analysis of the responses given to the items of the Social Appearance Anxiety Scale (SAAS) for the samples.

	Total Sample (*n* = 1495)						Male sample (*n* = 426)							Female sample (*n* = 1058)						
Item	*M*	*Md*	*Mo*	*SD*	*Sk*	*Ku*	*M*	*Md*	*Mo*	*SD*	*Sk*	*Ku*	*λ*	*M*	*Md*	*Mo*	*SD*	*Sk*	*Ku*	λ
1	2.91	3.00	3.00	1.10	0.29	−0.63	2.71	3.00	2.00	1.03	0.43	−0.43	0.59	2.98	3.00	3.00	1.12	0.23	−0.68	0.66
2	2.36	2.00	1.00	1.23	0.53	−0.71	1.97	2.00	1.00	1.07	0.85	−0.14	0.70	2.51	2.00	1.00	1.25	0.40	−0.85	0.74
3	2.57	2.00	2.00	1.25	0.37	−0.86	2.18	2.00	1.00	1.17	0.73	−0.38	0.74	2.72	3.00	2.00	1.25	0.25	−0.92	0.74
4	1.99	1.00	1.00	1.25	1.03	−0.12	1.85	1.00	1.00	1.16	1.18	0.26	0.87	2.05	2.00	1.00	1.28	0.96	−0.26	0.88
5	2.19	2.00	1.00	1.35	0.81	−0.65	1.77	1.00	1.00	1.08	1.34	0.91	0.88	2.36	2.00	1.00	1.41	0.61	−0.99	0.88
6	2.46	2.00	1.00	1.35	0.51	−0.95	2.24	2.00	1.00	1.28	0.75	−0.52	0.94	2.54	2.00	1.00	1.37	0.43	−1.06	0.96
7	2.38	2.00	1.00	1.33	0.58	−0.88	2.15	2.00	1.00	1.25	0.79	−0.53	0.94	2.47	2.00	1.00	1.35	0.49	−0.97	0.94
8	2.04	1.00	1.00	1.29	0.96	−0.29	1.93	1.00	1.00	1.22	1.06	−0.52	0.82	2.09	2.00	1.00	1.31	0.92	−0.37	0.85
9	1.78	1.00	1.00	1.17	1.42	0.94	1.67	1.00	1.00	1.06	1.59	1.58	0.82	1.81	1.00	1.00	1.21	1.37	0.75	0.81
10	1.82	1.00	1.00	1.15	1.26	0.55	1.61	1.00	1.00	0.98	1.60	1.79	0.88	1.90	1.00	1.00	1.20	1.14	0.19	0.86
11	2.44	2.00	1.00	1.32	0.52	−0.91	2.04	2.00	1.00	1.16	0.88	−0.25	0.86	2.61	2.00	1.00	1.34	0.38	−1.06	0.87
12	2.21	2.00	1.00	1.32	0.77	−0.65	1.79	1.00	1.00	1.12	1.36	0.91	0.91	2.38	2.00	1.00	1.36	0.59	−0.92	0.91
13	2.36	2.00	1.00	1.32	0.60	−0.84	1.98	2.00	1.00	1.14	1.00	0.42	0.93	2.51	2.00	1.00	1.36	0.45	−1.04	0.95
14	2.53	2.00	1.00	1.36	0.44	−1.06	2.01	2.00	1.00	1.16	0.97	−0.30	0.90	2.75	3.00	2.00	1.38	0.24	−1.21	0.91
15	2.10	2.00	1.00	1.33	0.90	−0.49	1.94	1.00	1.00	1.22	1.17	0.25	0.77	2.17	2.00	1.00	1.37	0.81	−0.70	0.81
16	2.18	2.00	1.00	1.27	0.80	−0.51	1.90	1.00	1.00	1.14	1.09	0.12	0.93	2.29	2.00	1.00	1.30	0.69	−0.68	0.93

*M*, Mean; *Md*, Median; *Mo*, Mode; *SD*, Standard Deviation; *Sk*, Skewness; *Ku*, Kurtosis; *λ*, Factor loading.

Table 3 presents the psychometric indicators of the SAAS for the men and women samples. The SAAS one-factor model showed adequate fit indices and factor loading of items (see Table 2) above 0.50 for both samples. In the convergent validity and reliability analyses, the values found for AVE, α, and ω were adequate for both samples (Table 3). Measurement invariance tests showed that the one-factor model of the SAAS presents strong equivalence between independent subsamples according to each gender, indicating its external validity (Table 3).

**Table 3:** Psychometric indicators of the Social Appearance Anxiety Scale (SAAS) for samples.

Psychometric indicators	Men (*n* = 426)	Women (*n* = 1058)
Comparative Fit Index (CFI)	0.979	0.979
Tucker-Lewis Index (TLI)	0.976	0.976
Standardized Root Mean Square Residual (SRMR)	0.043	0.044
Average Variance Extracted (AVE)	0.719	0.743
Ordinal alpha coefficient (α)	0.973	0.976
Omega coefficient (ω)	0.855	0.883
Correlation coefficient (*r*)^†^	0.113*	0.208*
Invariance (independent subsamples)^‡^	Subsamples *n* = 213 vs. *n* = 213	Subsamples *n* = 529 vs. *n* = 529
CFI (configural model)	0.982	0.980
CFI (metric model)	0.982	0.980
CFI (scalar model)	0.985	0.982
ΔCFI (metric - configural / scalar - metric)	0.000–0.003	0.000–0.002

^†^Correlation between the SAAS and Social Physique Anxiety Scale (SPAS). ^‡^Invariance test performed by the difference in CFI (ΔCFI). *statistical significance (*p* < 0.05).

About SPAS, the one-factor model presented adequate fit to the data of both samples of the present study (women: CFI = 0.984, TLI = 0.981, SRMR = 0.040, *λ* = 0.812–0.959, AVE = 0.783, *α* = 0.975, *ω* = 0.968; men: CFI = 0.985; TLI = 0.982; SRMR = 0.040; *λ* = 0.798–0.962; AVE = 0.783; *α* = 0.975; *ω* = 0.968). The correlation between SAAS and SPAS was 0.11 (*p* < 0.05) for men and 0.21 (*p* < 0.05) for women.

Table 4 presents the results of the structural models tested for each gender to verify the influence of individual characteristics on participants’ social appearance anxiety. For men, the variables income and how long they had been practicing physical exercise were not significantly related to social appearance anxiety. Therefore, these variables were excluded from the model. The refined model for men presented adequate fit to the data (CFI = 0.975, TLI = 0.981, SRMR = 0.048) and included the variables age, BMI, self-report of diagnosis of an eating disorder, and self-report of change in body appearance after the onset of the COVID-19 pandemic. Younger men, who had a higher BMI, self-reported a diagnosis of an eating disorder, or experienced perceived body changes after the onset of the COVID-19 pandemic presented higher levels of social appearance anxiety. For women, all independent variables tested were significant and the model presented an adequate fit to the data (CFI = 0.966, TLI = 0.979, SRMR = 0.051). Greater social appearance anxiety was observed among younger women, who had higher income, reported having started physical exercise more recently, reported having already received a diagnosis of an eating disorder, and perceived a change in their body after the onset of the COVID-19 pandemic.

**Table 4:** Structural model tested in the samples.

	Initial model^†^			Refined model^‡^		
Independent variable ➔ Dependent variable	*β*	*SE*	*p*	*β*	*SE*	*p*
Male sample (*n* = 426)						
Age ➔ Social Appearance Anxiety	−0.332	0.007	<0.001*	−0.345	0.006	<0.001*
Income ➔ Social Appearance Anxiety	−0.007	0.053	0.884	–	–	–
Body mass index➔ Social Appearance Anxiety	0.150	0.014	0.002*	0.158	0.014	0.001*
How long have been exercising ➔ Social Appearance Anxiety	−0.043	0.036	0.391	–	–	–
Diagnosis of eating disorder^#^ ➔ Social Appearance Anxiety	0.093	0.348	0.020*	0.092	0.347	0.022*
Change in body appearance after the pandemic^#^ ➔ Social Appearance Anxiety	0.129	0.049	0.008*	0.129	0.048	0.008*
Female sample (*n* = 1058)						
Age ➔ Social Appearance Anxiety	−0.297	0.004	<0.001*	–	–	–
Income ➔ Social Appearance Anxiety	0.067	0.032	0.020*	–	–	–
Body mass index➔ Social Appearance Anxiety	0.256	0.006	<0.001*	–	–	–
How long have been exercising ➔ Social Appearance Anxiety	−0.089	0.020	0.002*	–	–	–
Diagnosis of eating disorder^#^ ➔ Social Appearance Anxiety	0.214	0.106	<0.001*	–	–	–
Change in body appearance after the pandemic^#^ ➔ Social Appearance Anxiety	0.122	0.031	<0.001*	–	–	–

^†^Initial model indicates that all independent variables were tested simultaneously. ^‡^Refined model indicates that only the significant independent variables were included. *β*, standardized estimate; *SE*, standard error. *p < 0.05. #Self-reported measure.

## 4 Discussion

To the best of our knowledge, this is the first study that evaluated the psychometric properties of the Portuguese version of the SAAS one-factor model for men and women Brazilian adults who practice physical exercise. The study that translated and cross-culturally adapted the SAAS into Portuguese verified only its content validity and internal consistency (Donofre et al., 2021). Therefore, our study contributes to providing evidence on construct validity (i.e., factorial, convergent, and concurrent), measurement invariance in independent subsamples, and reliability. These data allow for verifying how the scale operates in assessing social appearance anxiety. Furthermore, we identified different individual characteristics for each gender that influenced the level of social appearance anxiety. Those results can be useful for clinical assessments, epidemiological protocols, and intervention studies, among others, aiming at improving people’s relationship with physical appearance and reducing social anxiety.

In factorial validity analysis of the SAAS one-factor model, excellent values for goodness-of-fit indices for both samples were obtained in CFA. A study conducted in Italy (Dakanalis et al., 2016) found similar results among adolescents from the community and those diagnosed with eating disorders. These results indicate that the SAAS one-factor model maintains a good fit across populations of distinct ages and clinical conditions. Still, the factor loadings of the 16 items of the SAAS were all adequate. Importantly, item 1 which is inverted in relation to the others presented adequate factor loading, but the values were low when compared to the other items. A study conducted in Belgium (Claes et al., 2012) also found similar results for women diagnosed with an eating disorder, with all factor loadings of the items being>0.70, except for item 1 which was 0.50. This can be explained by item wording, which being inverted seems to make it difficult for the reader to understand, but not to the point of demonstrating the need to remove it from the model.

We found adequate convergent validity of the SAAS one-factor model for the Brazilian data. As we did not find studies that used a similar methodology (i.e., AVE values), direct comparisons were not possible. In the present work, the convergent validity was analyzed from the factorial loadings of the items to investigate the extent of shared variance to assess the construct. This methodology was proposed by (Fornell and Larcker, 1981) and is recommended by specialized literature (Hair et al., 2019; Marôco, 2021).

The study that developed the SAAS (Hart et al., 2008) and other research works (Levinson and Rodebaugh, 2011; Claes et al., 2012; Dakanalis et al., 2016) examined the correlation between the scale and other measures of social anxiety, body image, and eating disorders, obtaining satisfactory results, with significant values for adequacy. To assess concurrent validity, we calculated the correlation between the SAAS and SPAS which, despite not being high, was statistically significant. A possible explanation for the low values of the correlations found in the present study is that despite the similarity between the concepts evaluated by each scale, they have a different construction. While the SPAS assesses social physique anxiety as a concern at the level only of the physical body and it does not include items assessing other domains of appearance-related anxiety (Hart et al., 2008) the SAAS investigates social appearance anxiety in different everyday contexts, such as in affective and professional life (Hart et al., 2008). The original study of the SAAS (Hart et al., 2008), also found a significant correlation (*r* = 0.59, *p* = 0.005) between the SAAS and SPAS for the total sample including men and women. This value in our study had a slightly smaller magnitude, and this disparity may be due to cultural issues, indicating that, in the Brazilian context, participants may have perceived the concepts captured by the SAAS and SPAS as different.

For reliability, we found adequate values for both genders (*α* and *ω* > 0.86), demonstrating that the SAAS one-factor model was able to produce consistent results to assess the latent concept (i.e., social appearance anxiety). These findings corroborate with the original study of the SAAS (Hart et al., 2008), as well as studies conducted in Turkey (Sahin and Topkaya, 2015) and Brazil (Donofre et al., 2021). In the measurement invariance tests, we found ΔCFI values < −0.01, indicating that the SAAS one-factor model was equivalent across independent subsamples evaluated within each gender, suggesting external validity, i.e., even under different conditions the factorial structure was maintained. A study conducted in Italy (Dakanalis et al., 2016) with adolescents also showed that the SAAS one-factor model was invariant across different groups, such as eating disorder present vs. eating disorder absent; girls vs. boys; and younger vs. older adolescents, among others. Although the Italian study did not analyze independent subsamples within each gender as we did, the results suggest that the SAAS one-factor model was equivalent across those different groups. Importantly, this does not mean that the groups share the same scores, but that the scale operates similarly between the groups or subsamples; therefore, comparisons between the groups are adequate. Future studies may focus on comparing social appearance anxiety scores between groups with characteristics like those of studies that guaranteed the measurement invariance.

Different relations were found in the structural model tested for men and women. Higher monthly income was associated with greater social appearance anxiety among those who identified as women. A study using the SAAS conducted in Turkey with university students to determine the relationship between social appearance anxiety and happiness in overweight girls found no significant difference between the average SAAS scores and the economic level of the participants (Unver et al., 2022). However, a study carried out with low-income women in southern Brazil found that body image dissatisfaction was higher among higher-income women (Kops et al., 2018). Thus, considering the differences between the concepts (i.e., social appearance anxiety and dissatisfaction with body image), it can be explained that they are different constructs that can overlap. Furthermore, a distinct conception of a beautiful or standard body can be found among women of different socioeconomic levels (Kops et al., 2018; Albuquerque et al., 2021), so these issues still need to be further explored. For men, income was not a significant predictor to influence social appearance anxiety. This may be associated with a social construction where those who identify as men and who have a higher income consider other aspects than physical appearance for success and social acceptance. Future studies should explore this hypothesis.

Among women, the more years that they have practiced physical exercise, the lower their social appearance anxiety. This result can be compared to that of a Canadian study carried out with people with obesity (Baillot et al., 2020), which found that participants with experienced lower levels of social physique anxiety had higher levels of moderate-to-vigorous physical activity. Moreover, physical exercise may positively influence the individual’s self-assessment of body image (Naivar Sen et al., 2020). A scoping review (Sabiston et al., 2018) pointed out that based on results and the most frequent published associations, participation in physical activity and sport was related to a more positive body image, without causal conclusions. However, there are also situations in which individuals experience social physique anxiety in environments where their bodies can be observed by other people. In this case, exercising can be an arduous task (Sabiston et al., 2018; Herring et al., 2021), so social physique anxiety can potentially hinder motivation for physical activity (Brunet and Sabiston, 2009). Therefore, not all people will adhere to the exercise in the same way or for similar reasons and it is necessary to develop individualized plans to understand the motives and underlying barriers (Campos et al., 2022).

Men did not exhibit a correlation between engagement in physical exercise and social appearance anxiety. A meta-analysis (Bassett-Gunter et al., 2017) evaluated the relationship between physical activity and body image specifically focusing on men. The study discussed that while social physique anxiety was significantly related to exercise in cross-sectional studies, similar results were not found in intervention studies. In this way, it is possible to reflect that the relief of symptoms of social physique anxiety was not considered a motivating mechanism for practicing physical exercise. Generally, men seek to practice physical exercises aiming at objective and/or perceptual body changes, such as the increase and definition of muscle mass, in addition to stimulating self-efficacy. Thus, the practice of physical exercise can to some extent contribute to the reduction of social anxiety levels with appearance, but this is not the driving reason to perform such activity.

In addition, to understand the disparities between men and women in findings related to the duration of their exercise habits and social appearance anxiety, it is important to consider physical activity as an integral component of a healthy lifestyle. A study using data from the *ELSA-Brasil* (Patrão et al., 2017) aimed to identify psychosocial factors associated with the adoption of a healthy lifestyle from a gender perspective. Four lifestyle indicators were examined, one of which was engagement in recreational physical exercise. The authors (Patrão et al., 2017) found that women generally led a healthier lifestyle compared to men. They also found that men who had housekeepers presented healthier dietary habits and engaged in more physical exercise. Furthermore, greater satisfaction with body image was associated with a healthier lifestyle among men, while this association was not observed among women (Patrão et al., 2017).

Comparing data from the *ELSA-Brasil* to a Portuguese cohort, a study found that Brazilian women tended to perceive themselves as lighter than their actual weight (Patrão et al., 2022). This perception was associated with various factors, including self-perceived poor health and a lack of physical activity (Patrão et al., 2022). This highlights body image-related issues, such as appearance anxiety, and physical exercise as components of lifestyle and health. Therefore, they can be understood and experienced differently across genders and influenced by the specific cultural and behavioral aspects of a population.

Despite the differences, some similarities have been observed in the structural models between men and women. Age was significantly related to social appearance anxiety in both genders, indicating that younger adults suffer from greater anxiety. This result can be explained by varied reasons, one of which is the greater exposure of younger people to social networks and the media. A Brazilian study (Sousa Silva et al., 2018) analyzed the imagery-discursive construction considering the impact of Instagram on the self-assessment of body image. The authors observed that the body image considered as “standard” in this network (i.e., thin and with prominent muscular definition) acts coercively on those who are outside the standards, generating a desire to rebuild the body and one’s own identity that can cause suffering and psychic exhaustion. Younger individuals, who are generally less able to neutralize emotions in situations that cause stress and discontent (Campos et al., 2020), when in contact with influences such as social media, may present greater social anxiety due to the appearance of the body.

Having a higher BMI significantly influenced to have greater social appearance anxiety for both genders. This corroborates other studies that used the SAAS (Radix et al., 2018). Another study found that SAAS did not significantly correlate with BMI but with body fat content (Levinson and Rodebaugh, 2011). These findings may suggest that people diagnosed as overweight or obese may suffer greater pressure from society and develop a state of anxiety. In both sexes, it is known that larger bodies due to a higher percentage of body fat are less desired and also stigmatized (Voges et al., 2019). Thus, further studies that assess the relationship between social appearance anxiety and body fat or BMI measurements are important in different samples, so that care strategies in favor of these people’s mental health can be developed later.

In both men and women, having already received a medical diagnosis of an eating disorder influenced the higher level of social appearance anxiety. The authors of the SAAS suggested that this scale should be evaluated in clinical contexts such as in patients who experience an eating disorder (Hart et al., 2008). Although we did not collect data on a clinical sample, some participants self-reported having been diagnosed with an eating disorder before the study, and these individuals were more susceptible to social appearance anxiety. Studies (Dakanalis et al., 2016; Radix et al., 2018) have shown that an eating disorder diagnosis can serve as a warning indicator for researchers when evaluating social appearance anxiety. This supports the importance of knowing the clinical history of an individual to develop more effective preventive or interventional approaches.

The report of a perceived change in appearance after the onset of the COVID-19 pandemic was significantly related to greater social appearance anxiety among both men and women. The social isolation to contain the advances of COVID-19 had its unquestionable importance, however, it exacerbated the situations of stress and anxiety among young adults in Brazil (Campos et al., 2020). Castro et al. (2021) identified in a Brazilian sample of resistance training practitioners that social distancing led to changes in different areas of the participants’ lives, including an increase in sedentary behavior, amount of sleep, and greater body image dissatisfaction, which is in line with the result of the present study. As the SAAS assesses an important concept related to the social context (Hart et al., 2008), it can be a useful tool to collect data that can support the development of care strategies in favor of physical and mental health.

Although this study has several strengths it also has limitations. First, the cross-sectional design of the study, which does not allow for establishing a cause-and-effect relationship between the variables investigated in the structural models. The second refers to the sample that mostly included women. Furthermore, the majority of participants are individuals from the Southeastern region of Brazil, whites, and with a high educational level, which does not allow generalization of the results to all Brazilian contexts. The third limitation is associated with the variables included in the structural models, as they were selected based on the literature and our experience, and this may have excluded characteristics considered by other researchers as relevant. Thus, we suggest that future research protocols consider these limitations to develop future studies.

Despite the mentioned limitations, the present study can contribute to the scientific and clinical communities’ understanding of social appearance anxiety from the use of a psychometric scale. The methodological strategies used to assess the psychometric properties of the SAAS can be useful in developing future research protocols that aim to study mental health and body image. Furthermore, the use of the SAAS becomes promising to investigate signs and symptoms that can cause psychological distress, being a relevant tool for professionals who work especially in the health area. Thus, the identification of social appearance anxiety can be measured in a valid and reliable way from the SAAS, contributing to the development of preventive and interventionist strategies. Finally, the results of the structural models indicate important characteristics elucidating groups vulnerable to experiencing social distress to their body appearance, serving as a warning indicator to professionals in the healthcare context.

## 5 Conclusion

The data obtained with the Portuguese version of the SAAS presented good indicators of validity and reliability when applied to Brazilian adult samples of both genders who practice physical exercise. Participants’ individual characteristics influenced the level of social appearance anxiety and were different between genders. Only women, those with higher income and who have started physical exercise more recently presented greater social appearance anxiety. For both men and women, younger age, higher BMI, having already been diagnosed with an eating disorder, and self-perception of body change after the onset of the COVID-19 pandemic were significant predictors of greater social appearance anxiety.

The satisfactory psychometric properties of the SAAS indicate its effectiveness in screening for signs of social appearance anxiety. Additionally, the various individual characteristics that influence this type of anxiety highlight the critical need to consider multiple factors when examining men and women. These findings can be valuable for future research protocols, prevention strategies, and mental health interventions.

## Data availability statement

The raw data supporting the conclusions of this article will be made available by the authors, without undue reservation.

## Ethics statement

The studies involving humans were approved by Research Ethics Committee of the Faculty of Pharmaceutical Sciences of Araraquara (UNESP). The studies were conducted in accordance with the local legislation and institutional requirements. The participants provided their written informed consent to participate in this study.

## Author contributions

GD: Conceptualization, Data curation, Formal analysis, Investigation, Validation, Visualization, Writing – original draft. JC: Conceptualization, Methodology, Visualization, Writing – review & editing. PS: Conceptualization, Investigation, Methodology, Writing – review & editing. JM: Investigation, Methodology, Software, Writing – review & editing. LC: Conceptualization, Funding acquisition, Methodology, Resources, Software, Visualization, Writing – review & editing. WS: Conceptualization, Investigation, Methodology, Project administration, Supervision, Writing – original draft.
